# Trends in In-Hospital Mortality in Patients Admitted With Cardiovascular Diseases in the United States With Demographics and Risk Factors of All Cardiovascular In-Hospital Mortality: Analysis of the 2021 National Inpatient Sample Database

**DOI:** 10.7759/cureus.70620

**Published:** 2024-10-01

**Authors:** Michael Morgan, Vikas Yellapu, Daryn Short, Cara Ruggeri

**Affiliations:** 1 Internal Medicine, St. Luke's Hospital, Bethlehem, USA; 2 Cardiology, St. Luke's University Health Network, Bethlehem, USA; 3 Medicine, Temple University, Philadelphia, USA; 4 Internal Medicine, St. Luke's University Health Network, Bethlehem, USA

**Keywords:** cardiovascular diseases, demographics, in-hospital mortality, nationwide inpatient sample (nis), risk factors

## Abstract

Introduction and background

Cardiovascular diseases (CVDs) encompass a range of disorders involving coronary artery diseases, valvular heart diseases, myocardial diseases, pericardial diseases, hypertensive heart diseases, heart failure (HF), and pulmonary artery diseases. Given the high prevalence of CVDs, understanding both overall and in-hospital mortality rates from these diseases is crucial. Unsurprisingly, most research, procedures, and new pharmacological interventions aim to reduce these rates. No recent studies have comprehensively detailed in-hospital mortality rates, demographics, and risk factors for all CVDs combined. Yet, in-hospital mortality rates due to CVD significantly impact patients' families and healthcare teams and serve as a critical measure of healthcare system development and effectiveness. Therefore, analyzing in-hospital mortality rates is essential for filling the gap in the recent comprehensive analysis of in-hospital mortality rates, demographics, and risk factors of all CVDs.

Method

The study used data from the National Inpatient Sample and the Nationwide Inpatient Sample (NIS) Databases of 2021 and HCUP tools. The NIS database extrapolates national estimates based on a stratified sample of 20% of US hospital discharges. Results were expressed as probability and relative risk using the t-test, with a P-value <0.05 being statistically significant. Statistical analyses were done using Stata statistical software version 18 (StataCorp LLC, College Station, TX, US).

Results

This study included 6,666,752 hospital admissions in the United States. Of these, 2,337,589 patients were admitted with CVDs and related symptoms, with 70,552 deaths occurring during hospitalization, resulting in an in-hospital mortality rate of 3.01% due to CVDs. Our study showed all CVD-induced in-hospital mortality combined was found to have a higher association with diabetes but a lower association with hypertension, hyperlipidemia, alcohol, and smoking.

Conclusion

The highest rates of cardiovascular disease in-hospital mortality are cardiac arrest, rupture of the cardiac wall as a complication of acute myocardial infarction, cardiogenic shock, rupture of papillary muscle as a complication of acute myocardial infarction, and rupture of chorda tendinea as a complication of acute myocardial infarction. The most common causes of CVD in-hospital mortality are non-ST-elevation myocardial infarction (NSTEMI) (19.20%), ST-elevation myocardial infarction (STEMI) (17.80%), cardiac arrest (15.10%), hypertensive heart disease with heart failure (12.50%), ventricular fibrillation (4.70%), ventricular tachycardia (3.30%), and aortic stenosis (2.10%). The most common risk factors for CVD in-hospital mortality are age, male gender, and diabetes. Proper diabetes control and management might be the highest preventive measure for all CVD-induced in-hospital mortality.

## Introduction

Cardiovascular diseases (CVDs) encompass a range of disorders involving coronary artery diseases, valvular heart diseases, myocardial diseases, pericardial diseases, hypertensive heart diseases, heart failure (HF), and pulmonary artery diseases. CVDs are the most common cause of death globally and in the United States. The World Health Organization has estimated that 12 million deaths occur worldwide every year due to heart diseases. The early prognosis of cardiovascular diseases can aid in making decisions on lifestyle changes in high-risk patients and reduce complications [1].

Over the last decades, although the age-standardized mortality rates of CVD declined by 27.3%, the number of deaths increased by 42.4% from 1990 to 2015. On the other hand, CVD led to over 17 million deaths, 330 million years of life lost, and 35.6 million years lived with disability in 2017 worldwide. Meanwhile, it was projected that CVD would be the cause of more than 23 million deaths in 2030 around the world [2].

Given the high prevalence of CVDs, understanding both overall and in-hospital mortality rates from these diseases is crucial. Unsurprisingly, most research, procedures, and new pharmacological interventions aim to reduce these rates. 

Most of the previous studies have focused on CVD mortality in general and not on CVD in-hospital mortality rates. Yet, in-hospital mortality rates due to CVD significantly impact patients' families and healthcare teams and serve as a critical measure of healthcare system development and effectiveness. Therefore, analyzing in-hospital mortality rates is essential for evaluating and comparing healthcare quality. 

Addressing patients with end-stage cardiovascular diseases through palliative care and goals of care discussions early can prevent the increase in in-hospital mortality due to cardiovascular diseases. It can reduce the number of unnecessary visits to the hospitals.

## Materials and methods

Data source

The study used data from the National Inpatient Sample and the Nationwide Inpatient Sample (NIS), Healthcare Cost and Utilization Project (HCUP), the Agency for Healthcare Research, Quality HCUP Databases of 2021, HCUP tools and products, including Clinical Classifications Software (CCS) or Clinical Classifications Software Refined (CCSR), Elixhauser Comorbidity Software (HCUP, AHRQ, Rockville, Maryland, US) and the HCUP-NIS 2016-2021 Diagnoses and Procedures Frequency Sheet. The NIS database extrapolates national estimates based on a stratified sample of 20% of US hospital discharges [3-6].

We have used the HCUP tools to obtain the different cardiovascular diseases with their ICD-10-CM codes reported under the NIS. Elixhauser Comorbidity Software was used to help show the associated comorbid conditions with each CVD.

Study population

The study included 2,337,589 patients with CVDs who were admitted to the United States hospital in 2021. The study also included 70,552 patients who died with CVDs during the hospitalization [3-6].

We have studied the rates and trends of in-hospital mortality of patients admitted to hospitals in the United States with cardiovascular diseases and symptoms in 2021. We have divided the cardiovascular diseases into groups, including valvular diseases, hypertension and hypertensive heart diseases, heart failure, cardiomyopathies, coronary artery diseases and their complications, intracardiac thrombosis, coronary artery dissection and aneurysm, pulmonary artery diseases, pericardial diseases, myocarditis, arrhythmias, thoracic aortic dissection and aneurysms, orthostatic hypotension, chest pain, and cardiogenic shock. All patients included in the study had one of the listed cardiovascular diagnoses as a primary diagnosis for hospital admission. Categorization was based on the NIS reporting and clinical relationship between diseases.

We have studied the rate of each CVD's in-hospital mortality compared to the total number of patients admitted with the same disease. We have also studied the risk factors in patients who died during hospitalization due to all CVDS combined by comparing them to the risk factors in patients who survived the hospitalizations with all CVDs combined.

Patient characteristics

We have studied demographics, including age, race, gender, hospital region, hospital location, and patient location. We classify the age as equal to or above 65 and below 65. The gender was classified into male and female. The race was classified into White, Black, Hispanic, Asian, Native American, and others, as reported under the NIS. Patient location was classified based on the counties with population numbers. We have also studied risk factors associated with hypertension, diabetes, hyperlipidemia, obesity, smoking, and alcohol use disorder, which were obtained from the comorbidity software under the NIS.

Outcome measures

The outcomes evaluated were in-hospital mortality, risk factors associated with in-hospital mortality, and associated comorbid conditions. We have used the ICD-10-CM coding system to identify patients admitted to the hospital in the USA with a primary cardiovascular diagnosis. We have identified the percentage of patients who had died during the admission to the total patients admitted with all cardiovascular diseases.

Statistical analysis

Weighted data were used to calculate the percentage of in-hospital mortality for individual cardiovascular diseases, and unweighted data were used to study the risk factors associated with in-hospital mortality. Results were expressed as probability and relative risk using the t-test to compare the risk factors between the two variables, which include patients who died during the hospitalization and patients who survived the hospitalizations. Statistical analyses were done using Stata statistical software version 18 (StataCorp LLC, College Station, TX, US). Stata tables were generated and included the different variables used in the study.

## Results

Some CVDs can have very high mortality rates. Still, they do not represent the most common causes of CVD in-hospital mortality due to the low incidence of these diseases. For example, the in-hospital mortality rate for patients with a rupture of the cardiac wall as a complication of acute myocardial infarction is 71.70%. However, it represents only 0.2% of the total CVD-induced in-hospital mortality. It is important to understand the difference between the most fatal and common causes of in-hospital mortality (Table 1) [3-6]. 

**Table 1:** Rates of in-hospital mortality in patients admitted with cardiovascular diseases and symptoms The study used data from the National Inpatient Sample and the Nationwide Inpatient Sample (NIS), Healthcare Cost and Utilization Project (HCUP), Agency for Healthcare Research, Quality HCUP Databases of 2021, HCUP tools and products including Clinical Classifications Software (CCS) or Clinical Classifications Software Refined (CCSR), Elixhauser Comorbidity Software and HCUP-NIS 2016-2021 Diagnoses and Procedures Frequency Sheet [3-6].

	Cardiovascular diseases	Total number of patients presented with each CVD	Total number of patients who died during hospitalization from each CVD	Percentage of patients who died during hospitalization to the number of patients presented with each CVD	Percentage of patients who died during hospitalization to the total number of patients died from all CVDS
Valvular diseases	Rheumatic mitral stenosis	1,115	75	6.7%	0.1%
	Rheumatic mitral stenosis with insufficiency	1,535	63	4.2%	0.08%
	Rheumatic disorders of both mitral and aortic valves	8,180	320	3.9%	0.4%
	Disorders of both mitral and tricuspid valves	4,770	140	2.9%	0.1%
	Comb rheumatic disorder of mitral, aortic and tricuspid valves	5,735	270	4.7%	0.3%
	Acute and subacute infective endocarditis	11,620	595	5.1%	0.8%
	Nonrheumatic mitral insufficiency	28,740	550	1.9%	0.7%
	Nonrheumatic mitral prolapse	1,920	25	1.3%	0.03%
	Nonrheumatic mitral stenosis	870	64	7.3%	0.08%
	Nonrheumatic aortic stenosis	94,425	1505	1.5%	2.1%
	Nonrheumatic aortic insufficiency	4,985	105	2.1%	0.1%
	Nonrheumatic aortic stenosis with insufficiency	10,860	260	2.3%	0.3%
HTN and hypertensive heart diseases	Essential hypertension	7,090	10	0.1%	0.01%
	Hypertensive heart disease with HF	468,864	9225	1.9%	12.5%
	Hypertensive heart disease without HF	1,095	15	1.3%	0.02%
	Hypertensive urgency	72,095	150	0.2%	0.2%
	Hypertensive emergency	65,350	180	0.2%	0.2%
Heart failure	Acute systolic heart failure	9,610	205	2.1%	0.2%
	Acute on chronic systolic heart failure	30,745	1175	3.8%	1.6%
	Acute diastolic heart failure	5,260	105	1.9%	0.1%
	Acute on chronic diastolic heart failure	21,665	775	3.5%	1.1%
	Acute combined systolic and diastolic heart failure	2,155	60	2.7%	0.08%
	Acute on chronic combined systolic and diastolic heart failure	15,060	595	3.9%	0.8%
	Acute right heart failure	520	45	8.6%	0.06%
	Acute on chronic right heart failure	1,375	130	9.4%	0.1%
	End-stage heart failure	605	120	19.8%	0.1%
Cardiomyopathies	Dilated cardiomyopathy	3,290	120	3.6%	0.1%
	Obstructive hypertrophic cardiomyopathy	3,420	50	1.4%	0.06%
	Alcoholic cardiomyopathy	590	20	3.3%	0.02%
	Cardiomyopathy, unspecified	1,450	85	5.8%	0.1%
	Ischemic cardiomyopathy	3,450	235	6.8%	0.3%
Coronary artery diseases and associated complications	Unstable angina	6,630	5	0.1%	0.006%
	Angina pectoris, unspecified	2,300	5	0.2%	0.006%
	ST elevation myocardial infarction	156,385	13055	8.3%	17.8%
	Non-ST elevation myocardial infarction	406,815	14125	3.4%	19.2%
	Myocardial infarction type 2	9,735	295	3%	0.4%
	Ventricular septal defect as current comp following AMI	125	35	28%	0.04%
	Thrombosis of atrium/auric append/ventricle as comp following AMI	110	5	4.5%	0.006%
	Takotsubo syndrome	9,110	210	2.3%	0.2%
	Rupture of card wall w/o hemopericardium as current comp following AMI	230	165	71.7%	0.2%
	Rupture of chorda tendineae as current comp following AMI	95	30	31.5%	0.04%
	Rupture of papillary muscle as current comp following AMI	185	75	40.54%	0.1%
	Post-infarction angina	1,735	5	0.2%	0.006%
Coronary artery dissection and aneurysm	Coronary artery aneurysm	175	10	5.7%	0.01%
	Coronary artery dissection	885	20	2.2%	0.02%
Pulmonary artery diseases	Primary pulmonary hypertension	1,225	85	6.9%	0.1%
	Pulmonary hypertension, unspecified	4,650	145	3.1%	0.1%
	Secondary pulmonary arterial hypertension	2,650	195	7.3%	0.2%
	Pulmonary hypertension due to left heart disease	855	60	7%	0.08%
	Pulmonary hypertension due to lung diseases and hypoxia	1,205	105	8.7%	0.1%
	Chronic thromboembolic pulmonary hypertension	935	65	5.3%	0.08%
	Chronic Cor pulmonale	620	20	3.2%	0.02%
	Chronic pulmonary embolism	875	10	1.1%	0.01%
	Aneurysm of pulmonary artery	165	10	6%	0.01%
Pericardial diseases	Infective pericarditis	1,880	15	0.7%	0.02%
	Acute pericarditis, unspecified	7,825	85	1%	0.1%
	Chronic constrictive pericarditis	730	30	4.1%	0.04%
	Hemopericardium, not elsewhere classified	375	45	12%	0.06%
	Pericardial effusion (noninflammatory)	21,310	750	3.5%	1%
	Post-cardiotomy syndrome	685	5	7.2%	0.006
Myocarditis	Infective myocarditis	1,420	15	1.1%	0.02%
	Acute myocarditis, unspecified	1,245	20	1.6%	0.02%
	Myocarditis, unspecified	2,545	15	0.5%	0.02%
Arrhythmias	Atrioventricular block, first degree	1,785	15	0.8%	0.02%
	Atrioventricular block, second degree	14,705	120	0.8%	0.1%
	Atrioventricular block, complete	42,880	1095	2.5%	1.4%
	Bifascicular block	965	15	1.5%	0.02%
	Long QT syndrome	600	30	5%	0.04%
	Cardiac arrest, cause unspecified	15,435	11115	72%	15.1%
	Supraventricular tachycardia	37,065	345	0.93%	0.4%
	Ventricular tachycardia	48,475	2430	5%	3.3%
	Atrial fibrillation	390,370	1525	0.3%	2%
	Atrial flutter	52,540	405	0.7%	0.5%
	Ventricular fibrillation	13,220	3490	26.3%	4.7%
	Sick sinus syndrome	38,020	335	0.8%	0.4%
Thoracic aortic dissection and aneurysm	Dissection of thoracic aorta	11,150	1335	11.9%	1.8%
	Thoracic aortic aneurysm, ruptured	815	200	24.5%	0.2%
	Thoracic aortic aneurysm, without rupture	10,085	230	2.2%	0.3%
Chest pain	Precordial pain	3,395	10	0.2%	0.01%
	Other chest pain	76,680	55	0.1%	0.07%
	Chest pain, unspecified	41,690	65	0.1%	0.08%
Cardiogenic shock	Cardiogenic shock	1,500	705	47%	0.9%
Intracardiac thrombosis	Intracardiac thrombosis	2,060	45	2.1%	0.06%

Our study showed that the highest in-hospital mortality rates were found in patients admitted with cardiac arrest (72%), rupture of the cardiac wall as a complication of acute myocardial infarction (71.70%), cardiogenic shock (47%), rupture of papillary muscle as a complication of acute myocardial infarction (40.54%), rupture of chorda tendineae as a complication of acute myocardial infarction (30.51%), ventricular septal defect as a complication of acute myocardial infarction (28%), ventricular fibrillation (26.30%), ST-elevation myocardial infarction (STEMI) involving the left main coronary artery (26.10%), ruptured thoracic aortic aneurysm (24.50%), end stage heart failure (19.80%), STEMI of unspecified site (13.50%), hemopericardium (12%), dissection of thoracic aorta (11.90%), STEMI involving other coronary artery of the anterior wall (9.70%), acute on chronic right heart failure (9.40%), pulmonary hypertension due to lung disease (8.70%), acute right heart failure (8.60%), STEMI involving the left anterior descending coronary artery (7.40%), and nonrheumatic valve mitral stenosis (7.30%) (Table 1) [3-6].

However, from the quantitative standpoint, the most common causes of in-hospital mortality from CVDs were non-ST-elevation myocardial infarction (NSTEMI) (19.20%), STEMI (17.80%), cardiac arrest (15.10%), hypertensive heart disease with heart failure (12.50%), ventricular fibrillation (4.70%), ventricular tachycardia (3.30%), and aortic stenosis (2.10%) of all CVD-induced in-hospital mortality (Table 1) [3-6].

The analysis of in-hospital mortality risk factors and demographics in patients admitted with all CVDs combined by comparing them to patients who survived the hospitalization where P-value <0.05 was statistically significant: most of these patients were aged 65 years or older (RR = 1.5185, P < 0.0001), male (RR = 1.0846, P <0.0001), and of Asian race (RR = 1.2857, P <0.0001). A significant proportion resided in not metropolitan or micropolitan counties (RR = 1.0734, P = 0.0192) and were admitted to urban teaching hospitals (RR = 1.1464, P < 0.0001), particularly in the western region (RR = 1.0939, P < 0.0001) (Table 2) [3-6].

**Table 2:** Demographics and risk factors of all CVDs in-hospital mortality combined compared to all patients admitted with CVDs The study used data from the National Inpatient Sample and the Nationwide Inpatient Sample (NIS), Healthcare Cost and Utilization Project (HCUP), Agency for Healthcare Research, Quality HCUP Databases of 2021, HCUP tools and products including Clinical Classifications Software (CCS) or Clinical Classifications Software Refined (CCSR), Elixhauser Comorbidity Software and HCUP-NIS 2016-2021 Diagnoses and Procedures Frequency Sheet [3-6].

Risk factors		Total number of patients admitted to the hospitals with CVDs	Number of patients who died during the hospitalization	Number of patients with all CVDs who survived the hospitalization	Probability	Relative risk	P-value
Age	≥65	289,866	10,338	279,528	3.50%	1.5185	<0.0001
	<65	172,605	4,054	168,551	2.30%	0.6586	<0.0001
Gender	Male	253,586	8,178	245,408	3.20%	1.0846	<0.0001
	Female	208,779	6,208	202,571	2.97%	0.922	<0.0001
Race	White	320,938	9,914	311,024	3.08%	0.9761	0.1749
	Black	65,958	1,725	64,231	2.60%	0.8186	<0.0001
	Hispanic	39,215	1,147	38,068	2.92%	0.9347	0.0259
	Asian or Pacific Islander	10,761	430	10,331	3.90%	1.2929	<0.0001
	Native American	2,359	72	2,284	3.05%	0.982	0.8756
	Other	11,254	472	10,782	4.19%	1.2857	<0.0001
Hospital location	Rural	36,195	1,037	35,158	2.86%	0.9145	0.0049
	Urban non-teaching	82,107	2,290	79,817	2.78%	0.8765	<0.0001
	Urban teaching	344,147	11,065	333,082	3.21%	1.1464	<0.0001
Hospital region	Northeast	85,551	2,607	82,944	3.04%	0.9746	0.2278
	Midwest	102,702	3,083	99,619	3.00%	0.9549	0.0213
	South	190,530	5,901	184,629	3.09%	0.9919	0.6277
	West	83,687	2,801	80,886	3.34%	1.0939	<0.0001
Patient location	Central counties of metro areas of ≥1 million population	122,291	3,793	118,498	3.10%	0.9952	0.7973
	Fringe counties of metro areas of ≥1 million population	114,344	3,290	111,054	2.87%	0.9021	<0.0001
	Counties in metro areas of 50,000-249,999 population	46,371	1,410	44,961	3.04%	0.9746	0.3513
	Counties in metro areas of 250,000-999,999 population	97,201	3,182	94,019	3.27%	1.0668	0.0011
	Micropolitan counties	44,891	1,481	43,410	3.29%	0.9697	0.2525
	Not metropolitan or micropolitan counties	34,618	1,150	33,468	3.32%	1.0734	0.0192
Smoking	Present	80,933	1,775	79,158	2.19%	0.6629	<0.0001
Alcohol	Present	20,720	609	20,111	2.93%	0.942	0.1432
Hyperlipidemia	Present	277,545	6,496	271,049	2.34%	0.5459	<0.0001
Diabetes	Present	169,028	5,456	163,572	3.22%	1.6073	<0.0001
Hypertension	Present	300,301	8,996	291,305	2.99%	0.8994	<0.0001

The highest incidence of CVD-induced in-hospital mortality among patients admitted to urban teaching hospitals can be explained by the complexity of cardiovascular cases either directly admitted or transferred to the teaching hospitals.

Our study showed that patients with all CVD-induced in-hospital mortality combined were found to have higher association with diabetes (RR = 1.6073, P < 0.0001) but lower association with hypertension (RR = 0.8994, P < 0.0001), hyperlipidemia (RR = 0.5459, P < 0.0001), alcohol (RR = 0.942, P = 0.1432), and smoking (RR = 0.6629, P < 0.0001) (Table 2) [3-6].

Despite hypertension, hyperlipidemia, and smoking being major risk factors for coronary artery diseases, they are not associated with CVD-induced in-hospital mortality due to the fact of combining all cardiovascular diseases as a common factor, which includes coronary and noncoronary diseases.

## Discussion

During the 20th century, there was a transition from communicable diseases to noncommunicable diseases as the most common causes of death. Public health and medical technologies dramatically reduced the communicable diseases ravages worldwide in little over a century by reducing involuntary exposure to pathogens (e.g., safer water, sewer, and food security systems; vector control), reducing the susceptibility of individuals to infection if exposed to the pathogen (e.g., immunization), and improving survivability among infected individuals (antibiotics) [7]. Currently, CVDs are non-communicable diseases and the leading cause of death worldwide [8-10].

The causes and risk factors of CVDs can be multifactorial, including genetics and lifestyle. Studies estimated that CVD heritability ranges between 40% and 50% [11-13]. Genetic testing can help in the early detection of CVDs [14]. Adequate lifestyle modification, such as physical activity, a healthy diet, weight loss, and smoking cessation, helps to reduce the risk of CVDs [15,16].

Individuals with preexisting or undiagnosed diseases who are at higher risk of subsequent morbidity or mortality may be more likely to be inactive. Therefore, the perceived benefits of exercise may merely represent the absence of such concomitant disease [17].

Diabetes is one of the most important risk factors for CVDs. It increases the risk for ischemic heart disease, and it causes neuropathy, retinopathy, and nephropathy, which worsens the morbidity of CVD patients. Diabetes is estimated as the sixth leading cause of disability worldwide [18]. Studies showed that one out of every two patients with diabetes is unaware of his disease, and even well-known diabetic patients might not be aware of the role of diabetes in CVD [19-21]. Diabetes control is more likely to reduce the CVD risk and mortality [22].

The Diabetes Control and Complications Trial (DCCT) showed a 41% risk reduction of cardiovascular events in type 1 diabetes. Moreover, during the post-trial nine-year follow-up observational period of the DCCT-Epidemiology of Diabetes Interventions and Complications (EDIC) trial, despite the loss of the original difference in HbA1c as a consequence of conventional treatment switching to an intensive approach and the less tight glycemic control in patients intensively treated, a risk reduction for any cardiovascular event (42%; P = 0.02) and for nonfatal myocardial infarction, stroke, or death for CVD (57%; P = 0.02) was fully achieved [23].

One of the new medications that help with controlling diabetes has shown an effect on cardiovascular protection and improving mortality in heart failure patients [24].

Frequent hospitalization can predict increased mortality risk. One of the most important measures for these patients with frequent hospitalization is the proper transition between hospital and home. Adequate care plans and close follow-up after discharge will help to reduce hospitalization and mortality [25]. Goals of care discussion and advanced care planning should be addressed early for patients with end-stage CVDs, like end-stage heart failure patients [26].

For example, in patients with HF, the use of 30-day rehospitalization as a healthcare metric and increased pressure to provide value-based care compel healthcare providers to improve efficiency and use an integrated care approach. Healthcare providers are using transition programs to achieve their goals. The comprehensive transition of care planning includes determining needs and resources in high-risk patients such as home health, palliative, or hospice care [27].

Medication nonadherence is another problem that might contribute to increased mortality in CVD patients [28]. Medication adherence should be addressed with every follow-up for these patients. Telehealth has played a role in patients' self-care adherence [29].

Studies showed a relationship between in-hospital mortality and nursing level of education [30]. According to Kalisch and Xie [31], missed nursing care is substantial, and similar levels are found in a number of hospitals. The reasons for missed nursing care in hospitals include staffing resources, material resources, and communication issues. The higher the staffing levels, the fewer occurrences of missed nursing care. Missed nursing care predicts adverse events (i.e., falls, pressure ulcers, new infections, and patient mortality) [31].

New and modern technologies, such as genetic testing and implantable devices, can help reduce the mortality risk from CVDs [32]. These implantable devices include pacemakers, defibrillators, and left ventricular assist devices [33].

Genetic tests can aid in establishing a diagnosis, guide medical management, and add to our understanding of inheritance patterns and for family counseling purposes. However, the utility of genetic testing depends on a multitude of factors, including but not limited to the specific condition, the patient's age, and the testing methodology [34].

Using modern technology in communication with patients can help reduce mortality. For example, sending text messages to patients with CVD can improve their control of risk factors [35].

Limitations

Some mild cardiovascular diseases (Table 1) with meager in-hospital mortality rates might not be even the primary cause of death for these patients, and their in-hospital mortality might be due to another pathology during the hospitalization course. For instance, in cases where patients with essential hypertension die during hospitalization, the cause of death likely differs from the primary diagnosis at admission. Also, some common CVD-induced in-hospital mortality can be secondary to a primary CVD that is not mentioned. For example, if patients were admitted with cardiac arrest and died during the hospitalization, it is probably secondary to a primary CVD that led to cardiac arrest and has not been mentioned.

## Conclusions

Our study showed that the most common causes of in-hospital mortality from CVDs were in patients admitted with NSTEMI (19.20%), STEMI (17.80%), cardiac arrest (15.10%), hypertensive heart disease with heart failure (12.50%), ventricular fibrillation (4.70%), ventricular tachycardia (3.30%), and aortic stenosis (2.10%) of all CVD-induced in-hospital mortality. However, the highest rates of CVD-induced in-hospital mortality are in patients admitted with cardiac arrest (72%), rupture of the cardiac wall as a complication of acute myocardial infarction (71.70%), cardiogenic shock (47%), rupture of papillary muscle as a complication of acute myocardial infarction (40.54%), rupture of chorda tendineae as a complication of acute myocardial infarction (30.51%), ventricular septal defect as a complication of acute myocardial infarction (28%), ventricular fibrillation (26.30%), STEMI involving the left main coronary artery (26.10%), ruptured thoracic aortic aneurysm (24.50%), end-stage heart failure (19.80%), STEMI of unspecified site (13.50%), hemopericardium (12%), dissection of thoracic aorta (11.90%), STEMI involving other coronary artery of the anterior wall (9.70%), acute on chronic right heart failure (9.40%), pulmonary hypertension due to lung disease (8.70%), acute right heart failure (8.60%), STEMI involving the left anterior descending coronary artery (7.40%), and nonrheumatic valve mitral stenosis (7.30%).

Demographically, the highest in-hospital mortality rates of all patients with CVD combined were observed in patients aged ≥ 65 years, predominantly Asian and male. The most common risk factor for all CVD-induced in-hospital mortality is diabetes.
